# Quantification of Biventricular Strains in Heart Failure With Preserved Ejection Fraction Patient Using Hyperelastic Warping Method

**DOI:** 10.3389/fphys.2018.01295

**Published:** 2018-09-19

**Authors:** Hua Zou, Ce Xi, Xiaodan Zhao, Angela S. Koh, Fei Gao, Yi Su, Ru-San Tan, John Allen, Lik Chuan Lee, Martin Genet, Liang Zhong

**Affiliations:** ^1^National Heart Centre Singapore, Singapore, Singapore; ^2^Department of Mechanical Engineering, Michigan State University, East Lansing, MI, United States; ^3^Duke-NUS Medical School, National University of Singapore, Singapore, Singapore; ^4^Institute of High Performance Computing, A^∗^STAR, Singapore, Singapore; ^5^Mechanics Department and Solid Mechanics Laboratory, École Polytechnique, C.N.R.S., Université Paris-Saclay, Palaiseau, France; ^6^M3DISIM Team, I.N.R.I.A, Université Paris-Saclay, Palaiseau, France

**Keywords:** heart failure with preserved ejection fraction, left ventricle, right ventricle, strain, hyperelastic warping

## Abstract

Heart failure (HF) imposes a major global health care burden on society and suffering on the individual. About 50% of HF patients have preserved ejection fraction (HFpEF). More intricate and comprehensive measurement-focused imaging of multiple strain components may aid in the diagnosis and elucidation of this disease. Here, we describe the development of a semi-automated hyperelastic warping method for rapid comprehensive assessment of biventricular circumferential, longitudinal, and radial strains that is physiological meaningful and reproducible. We recruited and performed cardiac magnetic resonance (CMR) imaging on 30 subjects [10 HFpEF, 10 HF with reduced ejection fraction patients (HFrEF) and 10 healthy controls]. In each subject, a three-dimensional heart model including left ventricle (LV), right ventricle (RV), and septum was reconstructed from CMR images. The hyperelastic warping method was used to reference the segmented model with the target images and biventricular circumferential, longitudinal, and radial strain–time curves were obtained. The peak systolic strains are then measured and analyzed in this study. Intra- and inter-observer reproducibility of the biventricular peak systolic strains was excellent with all ICCs > 0.92. LV peak systolic circumferential, longitudinal, and radial strain, respectively, exhibited a progressive decrease in magnitude from healthy control→HFpEF→HFrEF: control (-15.5 ± 1.90, -15.6 ± 2.06, 41.4 ± 12.2%); HFpEF (-9.37 ± 3.23, -11.3 ± 1.76, 22.8 ± 13.1%); HFrEF (-4.75 ± 2.74, -7.55 ± 1.75, 10.8 ± 4.61%). A similar progressive decrease in magnitude was observed for RV peak systolic circumferential, longitudinal and radial strain: control (-9.91 ± 2.25, -14.5 ± 2.63, 26.8 ± 7.16%); HFpEF (-7.38 ± 3.17, -12.0 ± 2.45, 21.5 ± 10.0%); HFrEF (-5.92 ± 3.13, -8.63 ± 2.79, 15.2 ± 6.33%). Furthermore, septum peak systolic circumferential, longitudinal, and radial strain magnitude decreased gradually from healthy control to HFrEF: control (-7.11 ± 1.81, 16.3 ± 3.23, 18.5 ± 8.64%); HFpEF (-6.11 ± 3.98, -13.4 ± 3.02, 12.5 ± 6.38%); HFrEF (-1.42 ± 1.36, -8.99 ± 2.96, 3.35 ± 2.95%). The ROC analysis indicated LV peak systolic circumferential strain to be the most sensitive marker for differentiating HFpEF from healthy controls. Our results suggest that the hyperelastic warping method with the CMR-derived strains may reveal subtle impairment in HF biventricular mechanics, in particular despite a “normal” ventricular ejection fraction in HFpEF.

## Introduction

Heart failure (HF) with preserved ejection fraction is a clinical syndrome in which patients have symptoms and signs of HF but normal or near-normal left ventricle ejection fraction (LVEF). Nearly 30–50% of patients worldwide with HF have HFpEF (Hogg et al., 2004), including Singapore (Zhong et al., 2013), and the prevalence appears to be rising. Based on large community and admission cohorts, some studies have suggested recently that the prognosis may not differ significantly between HFrEF and HFpEF patients, making HFpEF a substantially challenging public health issue with an increasing burden on the elderly population (Lo et al., 2013). Characterized by diastolic dysfunctions with increased LV stiffness, slow LV filling, and elevated LV end-diastolic pressure, HFpEF is most frequently associated with myocardial fibrosis or hypertrophy. Despite normal or nearly normal LVEF, ventricular contractility indexes used in both Western and Asian population indicate that systolic dysfunction is common in HFpEF patients (Borlaug et al., 2009; Zhong et al., 2011, 2013). Impaired LV systolic function may be revealed by measuring ventricular strain (Lo et al., 2013; Choudhary et al., 2016; Genet et al., 2016a).

In most studies, ventricular strain is measured using tissue doppler or 2-D speckle-tracking echocardiography (Flachskampf et al., 2015). However, tissue doppler-based assessment of LV longitudinal function is angle dependent and typically assesses only mitral annular motion (Koyama et al., 2003). Speckle-tracking is the most widely available imaging modality for quantitative assessment of the LV and RV structure and functions (Kraigher-Krainer et al., 2014); image quality of the RV is, however, often poor and somewhat subjective with quantitation accuracy limited by the complex chamber geometry (Haddad et al., 2008; De Siqueira et al., 2016). Cardiac magnetic resonance (CMR) imaging has emerged as the gold standard for quantitative assessment of LV and RV volumes and functions (Marcelo et al., 2016). It is superior to echocardiography for evaluating segmental wall motion abnormalities and extra cardiac findings due to its higher spatial resolution (Hussein et al., 2013). In addition, CMR was demonstrated to have superior reproducibility over two-dimensional (2D) echocardiography (Kleijn et al., 2012; Leng et al., 2015).

Interest in the RV function in HF arises from community-based studies showing that 83% of HFpEF patients have associated pulmonary hypertension and one-third of them have right ventricular dysfunction (Kanwar et al., 2016). These findings have generated interest to study RV function in HFpEF. However, very few studies have been undertaken to quantify motion in the RV myocardium and ventricular septum for the assessment of RV function and interaction between LV and RV. Here, we utilized a hyperelastic warping approach to quantify ventricular motion and function by estimating bi-ventricular strains from CMR images. The hyperelastic warping is a deformable image registration technique integrating finite deformation continuum mechanics with image-based data to obtain strain measurements from medical images such as MRI, positron emission tomography (PET), and computed tomography (CT; Rabbitt et al., 1995; Veress et al., 2002, 2013). In the hyperelastic warping method, a finite element (FE) model of the region of interest is deformed by a body force that depends on the difference in image intensities between the template and target images. Hyperelastic strain energy based on continuum mechanics is applied to constrain and regularize the deformation (Veress et al., 2005; Genet et al., 2015, 2016b). Note that other regularizers have also been proposed, such as incompressibility (Mansi et al., 2011), or equilibrium gap (Claire et al., 2004; Genet et al., 2018). Application of the hyperelastic warping approach in cardiac motion and function has focused primarily on quantifying LV strains (Veress et al., 2008, 2013; Genet et al., 2016b) that has been verified by tagged MRI (Phatak et al., 2009) and 3D CSPAMM MR images (Genet et al., 2015). This method has also been applied to quantify circumferential strain in individual patient with HFpEF (Zou et al., 2016) and pulmonary hypertension (Xi et al., 2016).

In this work, we performed further bi-ventricular strain measurement using the hyperelastic warping method to extract circumferential, longitudinal, and radial strains in three regions of the bi-ventricular unit, namely, LV, RV, and septum. The goals of this study are threefold: (i) develop a framework to simultaneously quantify circumferential, longitudinal, and radial strains in the bi-ventricle model, (ii) detect abnormalities in these three types of strains in HFpEF patients compared to HFrEF and normal controls, and (iii) study the inter- and intra-observer reproducibility of the hyperelastic warping approach in its application in HF patients.

## Materials and Methods

### Study Population

The HF patients were recruited from the Curvedness-based Imaging Study (CBIS), a prospective study initiated in 2012. The normal control subjects were recruited from the Cardiac Aging Study (CAS) (Koh et al., 2018), a prospective study initiated in 2014. Ten paired sub-groups of subjects were enrolled and underwent CMR scans. One control, one HFrEF patient, and one HFpEF patient were recruited for each sub-group. They were age-comparable and gender-matched. Normal controls had no known cardiovascular disease or other co-morbidities. Patients with a clinical history of HF were recruited as HF patients. Using 40% as an LVEF cut-off value, HF patients with LVEF > 40% were treated as HFpEF while those with LVEF < 40% as HFrEF. The studies were approved by the local Institutional Review Board, and all enrolled participants gave written informed consent. The demographics of the study groups are summarized in **Table 1**.

**Table 1:** Demographics of study populations.

Characteristics	Normal (n = 10)	HFpEF (n = 10)	HFrEF (n = 10)	HFpEF *versus* HFrEF§
Age (year)	52.1 ± 12.7	52.4 ± 12.5	52.7 ± 11.6	NS
Gender (F/M)	2/8	2/8	2/8	NS
Height (cm)	167.9 ± 8.6	164.1 ± 7.4	167.3 ± 10.8	NS
Weight (kg)	69.7 ± 11.4	76.5 ± 16.7	86.2 ± 22.2*	NS
BSA (m^2^)	1.79 ± 0.18	1.85 ± 0.23	1.97 ± 0.30	NS
SBP (mmHg)	133.7 ± 11.6	136.1 ± 34.1	124.2 ± 19.6	NS
DBP(mmHg)	81 ± 9.37	75.7 ± 25.8	71.9 ± 13.1	NS
LVEF (%)	65 ± 6	53 ± 7*	25 ± 9*	<0.05
LVEDV index (ml/m^2^)	71.0 ± 12.8	89.7 ± 17.0*	148.3 ± 53.1*	<0.05
LVESV index (ml/m^2^)	25.0 ± 6.6	43.1 ± 13.2*	114.0 ± 50.0*	<0.05
LVSV index (ml/m^2^)	46.1 ± 8.7	46.7 ± 7.4	34.3 ± 13.1	0.053
LV mass index (g/m^2^)	47.8 ± 6.6	69.1 ± 19.5*	79.3 ± 27.2*	NS
RVEF (%)	56 ± 6	57 ± 7	40 ± 12*	<0.05
RVEDV index (ml/m^2^)	79.5 ± 15.2	76.1 ± 16.7	89.6 ± 29.9	NS
RVESV index (ml/m^2^)	35.7 ± 9.4	33.1 ± 9.6	54.8 ± 26.3*	<0.05
RVSV index (g/m^2^)	43.7 ± 7.2	43.3 ± 9.7	34.7 ± 13.3	NS
Pulse (BPM)	72 ± 10	58 ± 10	83 ± 24	<0.05
NYHA (I), *n* (%)	N.A	3 (30)	4 (30)	NS
NYHA (II), *n* (%)	N.A	5 (50)	4 (30)	NS
NYHA (III), *n* (%)	N.A	1 (10)	2 (20)	NS
NYHA (IV), *n* (%)	N.A	1 (10)	0 (0)	NS
Atrial flutter/fibrillation, *n* (%)	N.A	2 (20)	1 (10)	NS
Cancer within last five years, *n* (%)	N.A	0 (0)	0 (0)	NS
Chronic renal insufficiency, *n* (%)	N.A	2 (20)	0 (0)	NS
Current smoker, *n* (%)	N.A	4 (40)	1 (10)	NS
Depression, *n* (%)	N.A	0 (0)	0 (0)	NS
Diabetes, *n* (%)	N.A	3 (30)	6 (60)	NS
Hyperlipidemia, *n* (%)	N.A	7 (70)	5 (50)	NS
Hypertension, *n* (%)	N.A	8 (80)	5 (50)	NS
Peripheral vascular disease, n (%)	N.A	1 (10)	0 (0)	NS
Myocardial infarction, *n* (%)	N.A	1 (10)	0 (0)	NS
Stroke, *n* (%)	N.A	0 (0)	1 (10)	NS
NTproBNP	N.A	2667.2 ± 2519.6	921.6 ± 837.7	<0.05


*Data are mean ± SD. BSA, body surface area; SBP, systolic blood pressure; DBP, diastolic blood pressure; LV, left ventricular; EF, ejection fraction; EDV, end-diastolic volume; ESV, end-systolic volume; SV, stroke volume; RV, right ventricular; HFrEF, heart failure with reduced EF; HFpEF, heart failure with preserved EF; NYHA, New York Heart Association; NTproBNP: N-terminal pro b-type natriuretic peptide. § Wilcoxon rank-sum test. ^∗^Statistically significant difference between HFpEF vs normal controls, HFrEF vs normal controls, Wilcoxon rank-sum test (p < 0.05).*

### CMR Image Acquisition

As part of the routine clinical protocol, HFpEF and HFrEF patients underwent CMR evaluation on a 3T system (Ingenia, Philips Healthcare, Netherlands) with a dStream Torso coil (maximal number of channels 32). The same imaging protocol was applied to the control subjects. Balanced turbo field echo (BTFE) end-expiratory breath hold cine images were acquired in multi-planner short-axis and long-axis views. The short-axis view included the images from the apex to basal. The long-axis images included the two-chamber, three-chamber, and four-chamber views. The following typical sequence parameters were used: TR/TE 3/1 ms, flip angle 45^o^, slice thickness 8 mm for short-axis, pixel bandwidth 1797 Hz, field of view 280–450 mm, temporal resolution ≈ 28 ms, in plane spatial resolution 0.6 mm × 0.6mm–1.1 mm × 1.1 mm, and frame rate was selected as 30 or 40 frames per cardiac cycle. Among these 30 subjects, 30 frames are used for all the short-axis view. For long-axis view, 26 subjects had 30 frames, and the other four had 40 frames.

### Framework to Obtain the Circumferential, Longitudinal, and Radial Strains

**FIGURE 1:**
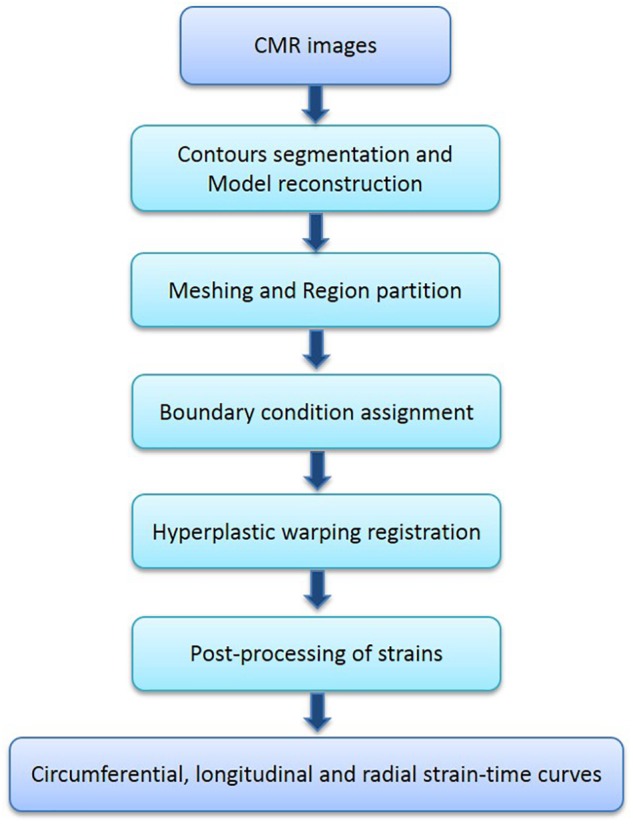
The overall workflow of strain measurement using hyperelastic warping method.

The strain acquisition framework was implemented using a combination of open-source software: MeVisLab (MeVis Medical Solution AG, Bremen, Germany), Gmsh (Geuzaine and Remacle, 2009), Fenics (Alnaes et al., 2015), and in-house code (Genet et al., 2014, 2015). The overall workflow is shown in **Figure 1**.

#### CMR Images

**FIGURE 2:**
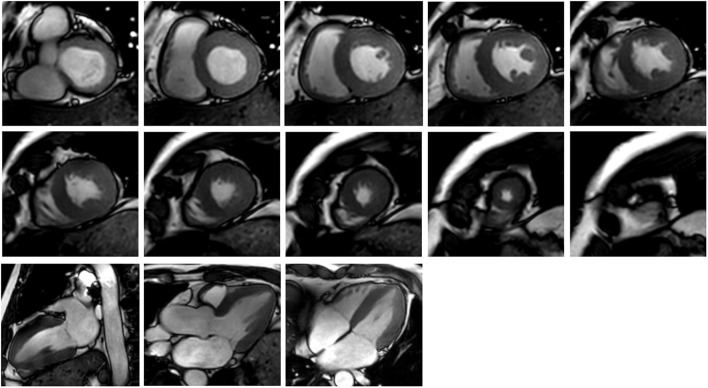
Cardiac MR images corresponding to 10 short-axis slices (the upper two rows) and three long-axis slices (the third row).

**Figure 2** shows an example of the short-axis and long-axis CMR images covering the LV and RV. They were used in contour segmentation and surface reconstruction, including short-axis images from basal to apex, and the four-chamber long-axis images.

#### Contours Segmentation and Model Reconstruction

**FIGURE 3:**
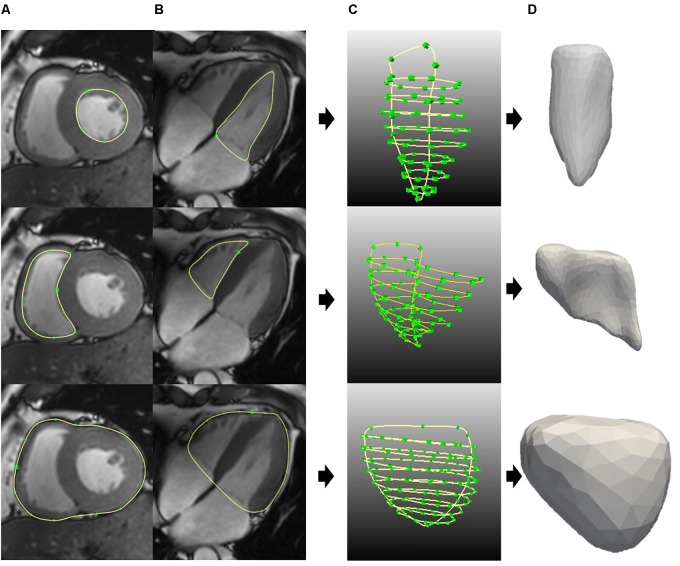
Generating the surface for LV endo (top), RV endo (middle), and bi-ventricular epicardia (bottom). **(A)** Short-axis contours segmentation. **(B)** Long-axis contours segmentation. **(C)** All the contours for surface generation. **(D)** LV endo, RV endo, and bi-ventricular epicardia surfaces.

**Figures 3A,B** show the contour segmentation from the short-axis and long-axis images for LV endocardium, RV endocardium, and bi-ventricular epicardium (from top to bottom, respectively). End of systole (ES) was chosen to be the time point to manually delineate the contours for model reconstruction – this is the cardiac phase when the aorta valve in three-chamber view starts to close. Depending on the size of the heart and the quality of the image, around 3–10 short-axis images and the four-chamber long-axis image were utilized. **Figures 3C,D** show, respectively, the contours used for surface reconstruction and the generated surfaces for the LV, RV, and bi-ventricular epicardium. Papillary muscles and trabeculated structures were not included as myocardium. After reconstruction, the model is corrected using a “WEMReducePolygons” module in Mevislab if there were mis-registration of the images.

#### FE Mesh Discretization and Region Partition

**FIGURE 4:**
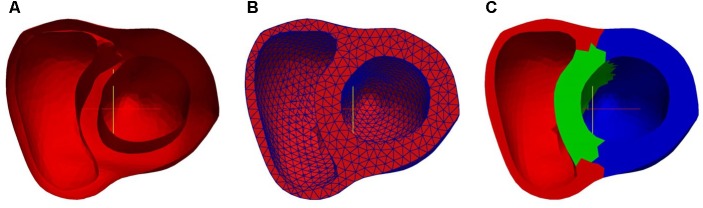
Bi-ventricle model: **(A)** 3D surface, **(B)** finite element mesh model, and **(C)** partition of region (red: RV free wall; green: septum; blue: LV free wall).

Following reconstruction of the three surfaces, they were assembled together to form the bi-ventricle model. **Figure 4A** shows the three-dimensional bi-ventricle model, and **Figure 4B** shows the FEmesh model generated by GMSH (Geuzaine and Remacle, 2009). Considering the computational time as well as the accuracy, we employed 0.3 as the mesh size. For these 30 cases, the number of FE nodes (points) ranged from 2286 to 3288 and the number of cells ranged from 7013 to 11,985. As previously mentioned, the model was partitioned into three regions (i.e., LV, RV, and septum) for strain study as shown in **Figure 4C**.

#### Boundary Conditions Assignment

**FIGURE 5:**
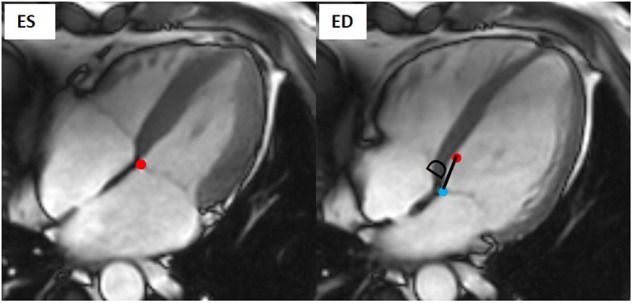
Measurement of septal displacement of septal in four-chamber view.

The short-axis images were used in the hyperelastic warping method. However, with the heart in motion, there was an excursion in the long-axis direction, thus a displacement was applied in the long-axis direction as a boundary condition in the hyperelastic warping method. The magnitude of the displacement was estimated by the mitral annular plane systolic excursion (MAPSE) at the septum measured in the four-chamber view in MeVisLab, as shown in **Figure 5**. During the dynamic deformation, the prescribed longitudinal displacement was controlled by a sine function.

#### Hyperelastic Warping Theory

Hyperelastic warping is a deformable image registration technique that can be used to measure cardiac strain derived from analysis of medical images such as MRI, ultrasound, and microPET imaging (Veress et al., 2013). In the hyperelastic warping method, a FE mesh (**Figure 4B**) was deformed along with a set of short-axis images during a cardiac cycle. The deformation of the FE mesh was defined as the mapping ϕ (*X*) = *X* + *u*(*X*), where *u* is the displacement field and *X* is the position. The deformation gradient was defined as

$$F(X)=\frac{\partial \phi }{\partial X}$$

The forces responsible for driving the registration deformation were derived from the difference in image intensity field between two volumetric image data sets by minimizing the following energy expression

$$E\left(\varphi \right)=\int W(X,C)\frac{dv}{J}-\int U(R(X)-T\left(\phi \right))\frac{dv}{J}$$

Here, *W* is the hyperelastic strain density energy function related to the material model of myocardium and C = F^T^F is the Cauchy–Green deformation tensor. A Neo–Hookean strain energy density function was used to define *W*, which is given as

$$W(X,C)=C_{1}\left(I_{1}-3\right)$$

where *C*_1_ is a material constant and *I*_1_ is the first invariant of the right Cauchy–Green deformation tensor. The energy term U produced an image force field responsible for the local registration of the discretized reference image *R* to the target image *T* and is expressed as

$$U(X,\phi )=\frac{\gamma }{2}(R{(X)-T\left(\phi \right))}^{2}$$

where γ is the penalty factor enforcing the alignment of the reference image to the target image. In summary, the FE mesh model was deformed to align with the target images via a computed image-based local body force term that depends on (1) the difference in image intensity between the template and target images, (2) the target image intensity gradient, and (3) a prescribed penalty factor.

**FIGURE 6:**
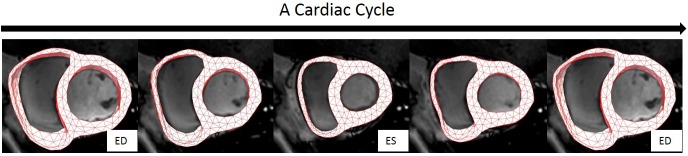
Registration of the bi-ventricular model with the CMR images during a cardiac cycle. Note: biventricular model was reconstructed only from the CMR images at ES.

The hyperelastic warping approach was implemented using FEniCS (Logg et al., 2012; Alnaes et al., 2015). The penalty parameter γ in Eq. 3 was set as 0.005 for all the cases. The method to optimize γ is referred to (Genet et al., 2017). **Figure 6** shows an example of the resultant deformation in the biventricular model from a normal subject computed from the hyperelastic warping method. As shown in the figure, the deformed biventricular model matched closely with the myocardium in CMR short-axis images at different cardiac time points.

#### Post-processing of Strains

The ES biventricular geometry was used as the initial configuration for tracking because we found that the image registration worked better when the myocardial wall at all subsequent time points is thinner than the initial one, which always revealed an image intensity gradient within the initial wall volume. Since the deformation gradient *F* was defined with ES as the initial configuration, the local Green-Lagrange strain tensor with end-diastole (ED) as the reference configuration – a more commonly used metric – was defined as

$$E=\frac{1}{2}\left(F^{T}F_{ED}^{-T}F_{ED}^{-1}-I\right)$$

In Eq. 5, *I* is the identity tensor and *F*_ED_ is the deformation gradient tensor at ED. Normal strains in the circumferential ε_CC_, longitudinal ε_LL_, and radial ε_RR_ directions were computed by projecting *E* onto these directions using ε_ii_ = *e_i_* ● *Ee_i_* with i ∈ (*C, L, R*). The circumferential *e_C_*, longitudinal *e_L_*, and radial *e_R_* were prescribed using a Laplace–Dirichlet rule-based (LDRB) algorithm (Bayer et al., 2012) with myofiber angle prescribed to be zero.

#### Strain–Time Curves

**FIGURE 7:**
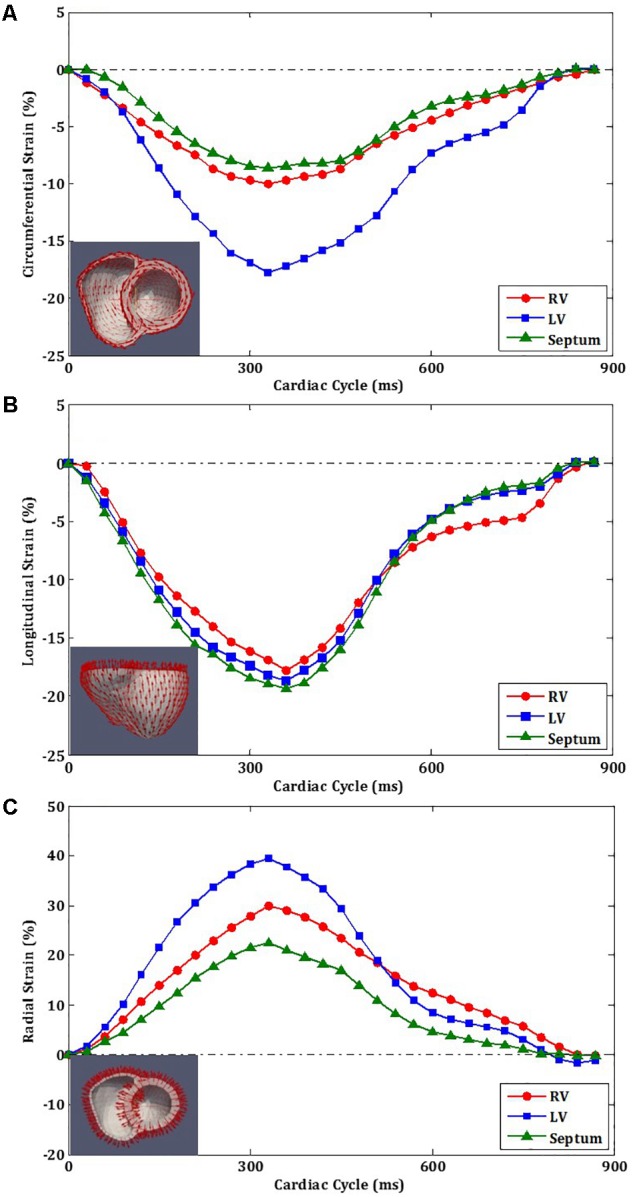
Strain orientation and strain–time curves of LV, RV, and septum for one cardiac cycle. **(A)** Circumferential strain. **(B)** Longitudinal strain. **(C)** Radial strain.

**Figure 7A** shows the circumferential direction assigned on the model and the circumferential strain-time curve, **Figure 7B** the longitudinal direction and longitudinal strain–time curve, and **Figure 7C** shows the radial direction and radial strain–time curve. These strain components were computed at each cardiac time point to construct the strain–time curves. The strain curves were computed by averaging the strains over all the elements with respect to the three regions: RV free wall, septum, and LV free wall as shown in **Figure 4C**. The effects of atrial contraction at late filling (i.e., “atrial kick”) are visible in the strain–time curves, particularly, in those associated with the LV.

### Reproducibility

Assessment of inter- and intra-observer variability of the hyperelastic warping method was performed on a random selection of nine cases: three controls, three HFpEF, and three HFrEF patients. Inter-observer variability was assessed by comparing measurements made by two independent observers from two different centers. Intra-observation variability was obtained from repeated measurements on these nine cases, 1 month apart, by the same observer.

### Statistical Analysis

Data were analyzed using SPSS (version 17.0, Chicago, IL, United States) and SAS (version 9.3, Cary, NC, United States). Comparisons of demographics, patient characteristics, and CMR measurements between patients and control subjects were performed using independent *t*-tests for normally distributed data, Mann–Whitney *U*-tests for non-normally distributed data, and Fisher’s exact tests for categorical data. Intra- and Inter-observer variability in peak circumferential, longitudinal, and radial strains were assessed by mean bias ± SD, limits of agreement, coefficient of variation (CV), and intra-class correlation coefficient (ICC) using data from nine randomly chosen subjects. ICC between 0.4 and 0.59 was considered fair, good between 0.60 and 0.74, and excellent when ≥0.75 (Aarsæther et al., 2012).

## Results

### Patient Demographics

Study subjects in each group consist of eight males and two females with a mean ± SD age of 52.1 ± 12.7, 52.4 ± 12.5, and 52.7 ± 11.6 years for controls, HFpEF, and HFrEF patients, respectively. Demographic and clinical characteristic of study subjects are given in **Table 1**. Compared with normal controls, LVEF was lower in both HF groups. Between the HF groups, LVEF was larger in HFpEF patients (53 ± 7%) than the HFrEF group (25 ± 9%). Both HF groups were comparable to controls with respect to height, BSA, SBP, DBP, LVSV index, RVEDV index, and RVSV index, but exhibited higher LVEDV index, LVESV index, and LV mass index than the controls (*p* < 0.05). The HFpEF patients had comparable RVEF and RVESV index to controls, while the HFrEF patients had lower RVEF and higher RVESV index than both controls and HFpEF patients (*p* < 0.05). HF groups were comparable relative to disease history, including NYHA class, atrial flutter/fibrillation, cancer within 5 years, chronic renal insufficiency, current smoker, depression, diabetes, hyperlipidemia, hypertension, peripheral vascular disease, myocardial infarction, and stroke. NTproBNP was much higher in HFpEF patients than in HFrEF patients (*p* < 0.05).

### Peak Systolic Circumferential, Longitudinal, and Radial Strains

**FIGURE 8:**
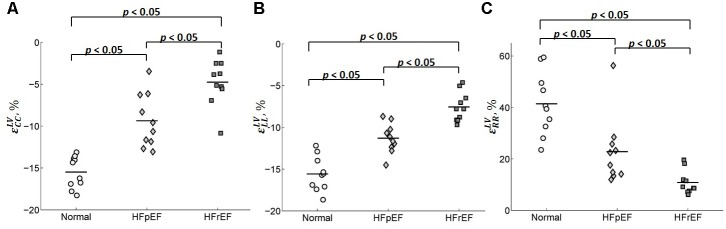
Scatter plot for the peak **(A)** circumferential, **(B)** longitudinal, and **(C)** radial strains for LV.

**Table 2** shows the average values of the peak circumferential, longitudinal, and radial strains in different regions (LV, RV, and septum) for control, HFpEF, and HFrEF patients. All the peak circumferential, longitudinal, and radial strains in the LV were, respectively, found to gradually decrease in magnitude (*p* < 0.05) from control → HFpEF→ HFrEF groups (circumferential: -15.5 ± 1.90, -9.37 ± 3.23, -4.75 ± 2.74; longitudinal: -15.6 ± 2.06, -11.3 ± 1.76, -7.55 ± 1.75; radial: 41.4 ± 12.2, 22.8 ± 13.1, 10.8 ± 4.61; **Table 2**). This may reveal impaired systolic LV function in both HF groups. **Figure 8** shows scatter plots for the three strains in the LV. Excellent separation of controls from both the HFpEF and HFrEF patients was observed in the peak circumferential strain (**Figure 8A**). Almost no overlap was found between the controls and the two HF groups of patients. Similar to the peak circumferential strain, peak longitudinal strain exhibited negligible overlap of controls with HFpEF and HFrEF (**Figure 8B**). Significant differences were found between the peak radial strain of normal controls and patients. However, there was some overlap between the normal controls and the patients (**Figure 8C**).

**Table 2:** Average circumferential, longitudinal, and radial strains for RV, LV, and septum.

Strain parameters	Normal	HFpEF	HFrEF	HFpEF vs. HFrEF§
$\varepsilon_{CC}^{RV}$ (%)	-9.91 ± 2.25	-7.38 ± 3.17	-5.92 ± 3.13*	NS
$\varepsilon_{CC}^{LV}$ (%)	-15.49 ± 1.90	-9.37 ± 3.23*	-4.75 ± 2.74*	<0.05
$\varepsilon_{CC}^{Sep}$ (%)	-7.11 ± 1.81	-6.11 ± 3.98*	-1.42 ± 1.36*	<0.05
$\varepsilon_{LL}^{RV}$ (%)	-14.49 ± 2.63	-12.04 ± 2.45*	-8.63 ± 2.79*	<0.05
$\varepsilon_{LL}^{LV}$ (%)	-15.58 ± 2.06	-11.30 ± 1.76*	-7.55 ± 1.75*	<0.05
$\varepsilon_{LL}^{Sep}$ (%)	-16.26 ± 3.23	-13.38 ± 3.02*	-8.89 ± 2.96*	<0.05
$\varepsilon_{RR}^{RV}$ (%)	26.79 ± 7.16	21.49 ± 10.01	15.15 ± 6.33*	NS
$\varepsilon_{RR}^{LV}$ (%)	41.41 ± 12.20	22.81 ± 13.05*	10.84 ± 4.61*	<0.05
$\varepsilon_{RR}^{Sep}$ (%)	18.51 ± 8.64	12.45 ± 6.38	3.35 ± 2.95*	<0.05


*Values are mean ± SD. $\varepsilon_{CC}^{RV}$, right ventricular peak circumferential strain; $\varepsilon_{CC}^{LV}$, left ventricular peak circumferential strain; $\varepsilon_{CC}^{Sep}$, septum peak circumferential strain; $\varepsilon_{LL}^{RV}$, right ventricular peak longitudinal strain; $\varepsilon_{LL}^{LV}$, left ventricular peak longitudinal strain; $\varepsilon_{LL}^{Sep}$, septum peak longitudinal strain; $\varepsilon_{RR}^{RV}$, right ventricular peak radial strain; $\varepsilon_{RR}^{LV}$, left ventricular peak radial strain; $\varepsilon_{RR}^{Sep}$, septum peak radial strain; HFrEF, heart failure with reduced ejection fraction; HFpEF, heart failure with preserved ejection fraction. § Wilcoxon rank-sum test. ^∗^Statistically significant difference between HFPEF vs. normal, HFREF vs. normal controls, Wilcoxon rank-sum test (*p* < 0.05).*

**FIGURE 9:**
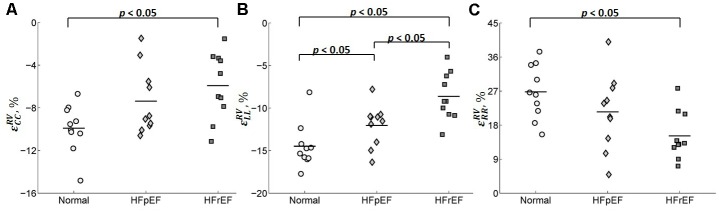
Scatter plot for the peak **(A)** circumferential, **(B)** longitudinal, and **(C)** radial strains for RV.

Circumferential and radial strains in RV were smaller in HFpEF patients compared with the Controls but no significant difference was observed (-9.91 ± 2.25 vs. -7.38 ± 3.17 for circumferential strain and 26.8 ± 7.16 vs. 21.5 ± 10.0 for radial strain). However, longitudinal strain in RV was significantly decreased in the HFpEF group when compared to the controls (-14.5 ± 2.63 vs. -12.0 ± 2.45; *p* < 0.05, **Table 2**). Significant differences were found for all the three strain components between HFrEF and normal controls (-5.92 ± 3.13 vs. -9.91 ± 2.25 for circumferential strain; -8.63 ± 2.79 vs. -14.5 ± 2.63 for longitudinal strain; and 15.2 ± 6.33 vs. 26.8 ± 7.16 for radial strain; all *p* < 0.05, **Table 2**). Only longitudinal strain was observed to differ significantly between HFrEF and HFpEF (-8.63 ± 2.79 vs. -12.04 ± 2.45; *p* < 0.05, **Table 2**). Scatter plots of RV strains are shown in **Figure 9**.

**FIGURE 10:**
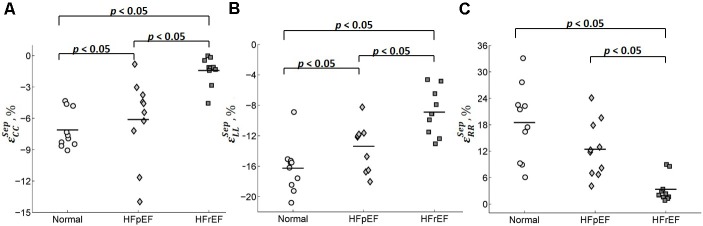
Scatter plot for peak **(A)** circumferential, **(B)** longitudinal, and **(C)** radial strains for septum.

Circumferential and longitudinal strains in the septum were depressed in HFpEF patients compared to the controls (-6.11 ± 3.98 vs. -7.11 ± 1.81 for circumferential strain; -13.4 ± 3.02 vs. -16.3 ± 3.23 for longitudinal strain; all *p* < 0.05, **Table 2**). Radial strain was smaller but not significantly different (12.5 ± 6.38 vs. 18.5 ± 8.64). Circumferential, longitudinal, and radial strains in the septum were all depressed in the HFrEF group compared with Controls (*p <* 0.05, **Table 2**). The scatter plot of strains for septum is shown in **Figure 10**.

### ROC Analysis and Cut-Off Values

**FIGURE 11:**
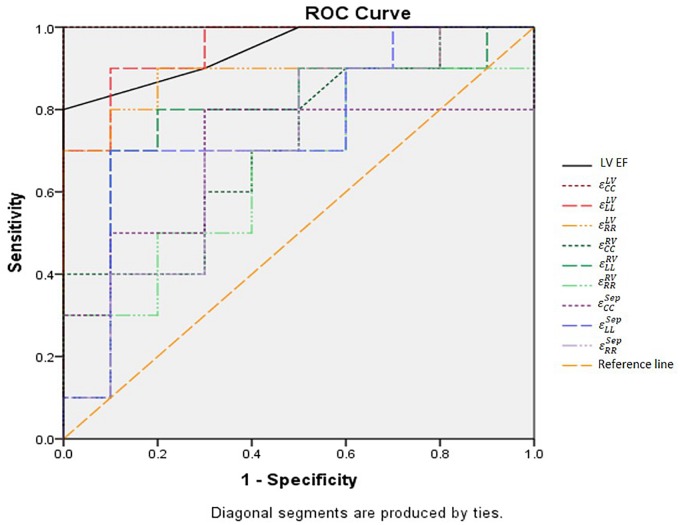
ROC curves of all strain parameters and LVEF for differentiating normal controls with HFpEF patients. LVEF, left ventricular ejection fraction; ε_CC_^RV^, right ventricular peak circumferential strain; ε_CC_^LV^, left ventricular peak circumferential strain; ε_CC_^Sep^, septum peak circumferential strain; ε_LL_^RV^, right ventricular peak longitudinal strain; ε_LL_^LV^, left ventricular peak longitudinal strain; ε_LL_^Sep^ : septum peak longitudinal strain; ε_RR_^RV^, right ventricular peak radial strain; ε_RR_^LV^, left ventricular peak radial strain; ε_RR_^Sep^, septum peak radial strain.

Receiver operating characteristic (ROC) curve analysis showed that LV circumferential and longitudinal strains were superior to the septal strain for differentiating normal controls from HFpEF patients (**Figure 11**). Area under the ROC curve (AUC) for LV circumferential strain was 1.00 with corresponding sensitivity and specificity of 1.00. AUC for LV longitudinal strain was 0.95 with sensitivity 0.90 and specificity 0.90 (**Table 3**).

**Table 3:** Sensitivity, specificity and AUC of the strains and LVEF for differentiating normal controls and HFpEF.

Parameters	Patient type	Cut-off value	Sensitivity	Specificity	AUC
$\varepsilon_{CC}^{RV}$ (%)	HFpEF	6.39	0.400	1.000	0.715
$\varepsilon_{CC}^{LV}$ (%)	HFpEF	13.10	1.000	1.000	1.000
$\varepsilon_{CC}^{Sep}$ (%)	HFpEF	7.26	0.800	0.700	0.690
$\varepsilon_{LL}^{RV}$ (%)	HFpEF	14.12	0.800	0.800	0.780
$\varepsilon_{LL}^{LV}$ (%)	HFpEF	12.85	0.900	0.900	0.950
$\varepsilon_{LL}^{Sep}$ (%)	HFpEF	14.90	0.700	0.900	0.750
$\varepsilon_{RR}^{RV}$ (%)	HFpEF	25.26	0.700	0.600	0.660
$\varepsilon_{RR}^{LV}$ (%)	HFpEF	30.53	0.900	0.800	0.890
$\varepsilon_{RR}^{Sep}$ (%)	HFpEF	20.51	0.900	0.500	0.700
LVEF	HFpEF	59	0.800	1.000	0.945


*$\varepsilon_{CC}^{RV}$, right ventricular peak circumferential strain; $\varepsilon_{CC}^{LV}$, left ventricular peak circumferential strain; $\varepsilon_{CC}^{Sep}$, septum peak circumferential strain; $\varepsilon_{LL}^{RV}$, right ventricular peak longitudinal strain; $\varepsilon_{LL}^{LV}$, left ventricular peak longitudinal strain; $\varepsilon_{LL}^{Sep}$, septum peak longitudinal strain; $\varepsilon_{RR}^{RV}$, right ventricular peak radial strain; $\varepsilon_{RR}^{LV}$, left ventricular peak radial strain; $\varepsilon_{RR}^{Sep}$, septum peak radial strain; LVEF, left ventricular ejection fraction.*

### Reproducibility

**Table 4** shows both intra- and inter-observer variability for nine randomly chosen cases (three normal controls, three HFpEF, and three HFrEF). In the Bland–Altman analysis, peak circumferential strain for LV had the best intra-observer agreement (bias, 0.08 ± 0.63; 95% CI, -1.16 to 1.32) and inter-observer agreement (bias, 0.67 ± 0.90; 95% CI, -1.10 to 2.45). Peak radial strain RV exhibited the largest intra-observer variability (bias, 1.28 ± 4.23; 95% CI, -7.01 to 9.57) and peak radial strain for LV had the largest inter-observer variability (bias, 5.6 ± 8.30; 95% CI, -10.63 to 21.9). All parameters had an excellent intra- and inter-observer agreement (ICC ≥ 0.92).

**Table 4:** Inter- and intra-observer agreement for nine randomly chosen cases (three control, three HFpEF, and three HFrEF).

Variable	Variability	Mean bias ±*SD*	Limits of agreement	Coefficient of variation (%)	ICC (95% CI)
$\varepsilon_{CC}^{RV}$	Intra-observer	0.20 ± 1.31	-2.38 to 2.78	10.40	0.954 (0.799, 0.990)
	Inter-observer	1.00 ± 1.24	-1.43 to 3.43	11.89	0.964 (0.839, 0.992)
$\varepsilon_{CC}^{LV}$	Intra-observer	0.08 ± 0.63	-1.16 to 1.32	4.18	0.997 (0.988, 0.999)
	Inter-observer	0.67 ± 0.90	-1.10 to 2.45	7.27	0.994 (0.973, 0.999)
$\varepsilon_{CC}^{Sep}$	Intra-observer	0.56 ± 0.51	-0.45 to 1.58	11.27	0.989 (0.843, 0.998)
	Inter-observer	0.55 ± 1.73	-2.85 to 3.95	23.35	0.952 (0.787, 0.989)
$\varepsilon_{LL}^{RV}$	Intra-observer	-0.91 ± 1.56	-3.97 to 2.15	9.30	0.940 (0.728, 0.987)
	Inter-observer	-0.27 ± 1.57	-3.35 to 2.81	8.48	0.945 (0.758, 0.988)
$\varepsilon_{LL}^{LV}$	Intra-observer	-0.54 ± 0.86	-2.23 to 1.14	5.77	0.989 (0.944, 0.998)
	Inter-observer	-0.56 ± 1.42	-3.35 to 2.23	8.98	0.974 (0.883, 0.994)
$\varepsilon_{LL}^{Sep}$	Intra-observer	-1.23 ± 1.35	-3.89 to 1.42	9.11	0.963 (0.692, 0.993)
	Inter-observer	-1.32 ± 2.38	-6.00 to 3.35	14.78	0.921 (0.649, 0.982)
$\varepsilon_{RR}^{RV}$	Intra-observer	1.28 ± 4.23	-7.01 to 9.57	12.41	0.953 (0.805, 0.989)
	Inter-observer	4.97 ± 3.52	-1.94 to 11.88	20.39	0.953 (0.794, 0.990)
$\varepsilon_{RR}^{LV}$	Intra-observer	0.47 ± 2.47	-4.36 to 5.31	6.15	0.997 (0.987, 0.999)
	Inter-observer	5.63 ± 8.30	-10.63 to 21.90	28.09	0.948 (0.769, 0.988)
$\varepsilon_{RR}^{Sep}$	Intra-observer	-0.87 ± 1.12	-3.06 to 1.31	8.74	0.995 (0.965, 0.999)
	Inter-observer	1.00 ± 2.29	-3.49 to 5.50	15.33	0.986 (0.939, 0.977)


*$\varepsilon_{CC}^{RV}$, right ventricular peak circumferential strain; $\varepsilon_{CC}^{LV}$, left ventricular peak circumferential strain; $\varepsilon_{CC}^{Sep}$, septum peak circumferential strain; $\varepsilon_{LL}^{RV}$, right ventricular peak longitudinal strain; $\varepsilon_{LL}^{LV}$, left ventricular peak longitudinal strain; $\varepsilon_{LL}^{Sep}$, septum peak longitudinal strain; $\varepsilon_{RR}^{RV}$, right ventricular peak radial strain; $\varepsilon_{RR}^{LV}$, left ventricular peak radial strain; $\varepsilon_{RR}^{Sep}$, septum peak radial strain.*

## Discussion

In this study, we compared myocardial strains estimated using a hyperelastic warping approach in the control, HFpEF, and HFrEF patients that are comparable in age and gender. To the best of our knowledge, this research work is the first to study biventricular three-dimensional strain (longitudinal, circumferential, and radial) based on CMR images in HF patients using the hyperelastic warping method. The major contributions of our study are as follows: (1) development of a novel framework for assessment of the biventricular mechanics of HF from CMR and (2) implementation of a viable and reproducible hyperelastic warping method for simultaneous evaluation of 3D circumferential, longitudinal, and radial strains for HF patients. The key findings from our study are as follows: (1) strains estimated in cine CMR images of HF patients using the hyperelastic are feasible and reproducible, (2) peak (absolute) circumferential, longitudinal, and radial strains in the RV, LV, and septum are highest in the normal controls followed by HFpEF to HFrEF patients, and (3) peak LV circumferential and longitudinal strain can better differentiate HFpEF patients from healthy subjects. These findings may provide a new method for simultaneous assessment of 3D biventricular strains in HF patients.

### LV Strain

We have found that all the three strain components (circumferential, longitudinal, and radial strains) in HFpEF and HFrEF patients were decreased compared to the normal subjects. Similar conclusions are also found in Yip et al. (2011), where they found a similar trend in global 2D circumferential, radial, and longitudinal strain (as well as torsion) using standard 2D Doppler and speckle-tracking echocardiography. Several studies (MacIver and Townsend, 2007; MacIver, 2008, 2011; Maciver et al., 2015) have used mathematical modeling to explain the apparent paradox of a reduction in longitudinal, circumferential, and radial strain but with a preserved LVEF. These studies suggest that the normal ejection fraction in patients with HF can be explained by the presence of left ventricular hypertrophy, which is found in the HFpEF patients of this study (LV mass index: 47.8 ± 6.6 g/m^2^ for normal controls vs. 69.1 ± 19.5 g/m^2^ for HFpEF, *p* < 0.05, **Table 1**).

In the literature, decreasing longitudinal strain was found in HFpEF patients (Borlaug, 2014). Specifically, a high prevalence of patients hospitalized with acute HFpEF with abnormal LV longitudinal strain suggests the presence of some previously unrecognized myocardial systolic dysfunction associated with this disease (Buggey et al., 2017). Consistent with our findings, a clinical trial including 219 HFpEF patients also demonstrated that LV longitudinal and circumferential strains are significantly lower in HFpEF patients when compared with normal controls (Kraigher-Krainer et al., 2014). Peak global longitudinal strain and strain rate in HFpEF patients are also found to be higher than those found in HFrEF patients (Carluccio et al., 2011). However, reports are conflicted with respect to the peak LV circumferential and radial strains in HFpEF patients. Some investigators suggest that reduced peak longitudinal strain in the presence of normal LVEF in HFpEF patients is due to a compensatory increase in circumferential and/or radial function (Fang et al., 2004; Paulus et al., 2007; Edvardsen and Haugaa, 2011; Vitarelli et al., 2015). Others suggest that peak radial strain in LV is increased in asymptomatic mildly hypertensive patients but decreases as LV hypertrophy (LVH) progresses and the severity of HF increases. Longitudinal and radial strains in the LV were reduced, but circumferential deformation and twist were normal in HFpEF patients in a study by Wang et al. (2008). To the contrary, longitudinal, radial, and circumferential deformation and twist are consistently reduced in patients with HFrEF.

### RV Strain

Right ventricular systolic dysfunction is a common feature in HFrEF that is associated with impaired functional capacity and portended a poor prognosis (Mohammed et al., 2014). The prevalence as well as the functional and prognostic implications of RV dysfunction in HFpEF are, however, less clear. Here, although there are no significant differences in all RV functional parameters (e.g., RVEF, RVEDV index, RVESV index, and RVSV index) between HFpEF patients and normal controls – see **Table 1**, we found that the peak RV longitudinal strain is significantly decreased in HFpEF patients compared with normal controls. This finding suggests that RV function may be impaired in HFpEF patients, and peak RV longitudinal strain may be a useful in detecting this change. Consistent with previous findings, all the three RV strain components are significantly reduced in HFrEF patients. This is also consistent with the significant difference in RVEF and RVESV index between HFrEF patients and normal subjects.

Systematic assessment of RV function is a widely recognized challenge owing to: (1) its complex geometry, (2) the limited definition of the RV endocardial surface occasioned by trabeculated myocardium, and (3) the retrosternal position of the RV that limits echocardiographic imaging windows (Cameli et al., 2014). It is, however, also becoming increasingly clear that assessing RV strain is important in analyzing HF. Meris et al. (2010) found that RV strain accurately identified reduced global RV function. Moreover, there is also mounting evidence that pulmonary hypertension with RV dysfunction is associated with a reduced regional longitudinal strain. A large body of data showing that pulmonary hypertension and RV dysfunction are also common in HFpEF (Gorter et al., 2016). However, the focus is on tricuspid annular plane systolic excursion (TAPSE), fractional area change (FAC), and tricuspid annular systolic velocity (RV S; Melenovsky et al., 2014; Leng et al., 2016). Studies reporting on RV strain, in particular those using CMR, are scarce. Our study on the evaluation of the longitudinal, circumferential, and radial strains in the RV suggests that hyperelastic warping method may be helpful.

### Septum Strain

Septum shape and deformation (i.e., area strain) has been studied in repaired tetralogy of Fallot patients with volume overloading (Zhong et al., 2012). However, septum strains by using warping method were investigated for the first time in HFpEF. The results revealed that circumferential and longitudinal strains decreased gradually from controls→HFpEF→HFrEF. The observed decrease in radial strain was approximately 50% for HFpEF compared to controls. However, due to the wide band of radial strain exhibited in the normal controls, the difference was not statistically significant. We emphasize that longitudinal strain was reduced in the septal region, as well as in the LV and RV.

### Reproducibility

Overall, LV strains have better reproducibility than the septum and RV, which is expected due to its thicker wall. Circumferential and longitudinal strains have excellent intra- and inter-observer agreement, although this is less so for radial strains that still possesses acceptable reproducibility. Peak LV circumferential strain has the best reproducibility, followed by peak LV longitudinal strain. On the other hand, peak radial strain has the worst reproducibility.

### Comparability of Strain Values to Other Published Results

Absolute peak strains obtained here appear to be smaller compared with previous studies. In normal subjects, peak LV circumferential, longitudinal and radial strains were -18.4 ± 2.9%, -19.1 ± 4.1%, and 39.8 ± 8.3% for Western population (Taylor et al., 2015); and -24.3 ± 3.1%, -22.4 ± 2.9%, and 79.0 ± 19.4% for Chinese population (Peng et al., 2018), respectively using CMR feature tracking. Comparing the peak values, those found here are relative smaller in the circumferential (-15.5 ± 1.90%) and longitudinal directions (-15.6 ± 2.06%), but slightly different in the radial direction (41.4 ± 12.2%). This disparity may be explained by a difference in strain definition used in that and our studies. Specifically, we have used Green–Lagrange strain that expressed as $\frac{\Delta L}{L}+\frac{1}{2}{\left(\frac{\Delta L}{L}\right)}^{2}$ in the one-dimensional case whereas Biot strain, reduced to $\frac{\Delta L}{L}$ in one-dimension was used in previous study. The additional term $\frac{1}{2}{\left(\frac{\Delta L}{L}\right)}^{2}$ leads to the Green–Lagrange strain having a lower peak value in the shortening (circumferential and longitudinal) directions and a larger peak value in the lengthening (radial) direction during systole. The disparity in strain estimated from feature tracking technique and deformable registration method was also discussed previously (Mangion et al., 2016).

### Limitations

First, to address the poor out-of-plane tracking at the ventricular base that arises because of the large out-of-plane resolution in the short-axis clinical CMR images, we have imposed a basal longitudinal displacement that varies sinusoidally with time. Despite able to obtain reasonable results even with this assumption, having a higher out-of-plane resolution (smaller slice thickness) may obviate the need to impose such an assumption.

Second, sample size in this study is relatively small. A larger sample size will be used in future studies to the increase statistical power.

## Conclusion

An advanced image registration method based on continuum mechanics was used to estimate three-dimensional peak circumferential, longitudinal, and radial strain in the bi-ventricular model. By dividing the biventricular unit into LV, RV, and septum, a new perspective was introduced for investigating strain in HFpEF and HFrEF and for studying the physiology of HFpEF disease. Diminishing magnitude in strain components from controls, HFpEF to HFrEF demonstrated subtle functional impairment in the LV and RV in HFpEF patients.

## Ethics Statement

The study was approved by the SingHealth Centralised Institutional Review Board, and written consent forms were obtained from all participants.

## Author Contributions

HZ and LZ contributed to the conception of the hypothesis of the study, implementation of all the analysis, and were involved in the evaluation of the results and preparation of the manuscript. CX contributed to the development of the code and inter-observer variability. XZ contributed to data preparation and analysis. AK and R-ST contributed to patient recruitment and image acquisition. FG and JA contributed to data analysis and statistics. LCL contributed to development of the code and critically revising of the work. MG contributed to development of the code. YS contributed to the evaluation of the results and preparation of the manuscript.

## Conflict of Interest Statement

The authors declare that the research was conducted in the absence of any commercial or financial relationships that could be construed as a potential conflict of interest.

## Abbreviations

EF: ejection fraction
HFpEF: heart failure with preserved ejection fraction
HFrEF: heart failure with reduced ejection fraction
LV: left ventricle
$\varepsilon_{CC}^{LV}$: left ventricular peak systolic circumferential strain
$\varepsilon_{LL}^{LV}$: left ventricular peak systolic longitudinal strain
$\varepsilon_{RR}^{LV}$: left ventricular peak systolic radial strain
MRI: magnetic resonance imaging
RV: right ventricle
$\varepsilon_{CC}^{RV}$: right ventricular peak systolic circumferential strain
$\varepsilon_{LL}^{RV}$: right ventricular peak systolic longitudinal strain
$\varepsilon_{RR}^{RV}$: right ventricular peak systolic radial strain
$\varepsilon_{CC}^{Sep}$: septum peak systolic circumferential strain
$\varepsilon_{LL}^{Sep}$: septum peak systolic longitudinal strain
$\varepsilon_{RR}^{Sep}$: septum peak systolic radial strain
